# Educational offer in older adult home care for migrant family care assistants: results from a multiple qualitative case study

**DOI:** 10.3389/fpubh.2025.1621071

**Published:** 2025-09-12

**Authors:** Sara Santini, Vanessa Frontalini, Georgia Casanova, Serena Cancellieri, Daniela Grignoli, Antonella Golino, Veronica Moretti, Riccardo Pronzato, Ilaria Chirico, Rabih Chattat

**Affiliations:** ^1^IRCCS INRCA National Institute of Health and Science on Ageing, Centre for Socio-Economic Research on Aging, Ancona, Italy; ^2^University of Molise, Campobasso, Italy; ^3^University of Bologna, Bologna, Italy

**Keywords:** migrant care worker, migrant family care assistants, older adult care training, education in older adult care, older people with long-term needs

## Abstract

**Introduction:**

The increasing number of older people with multiple chronic diseases determines care demand, while the number of formal and informal caregivers is decreasing. In many EU countries, migrant family care assistants (MFCAs) bridge the gap between supply and demand of H24 tailored home care, often without training in older adult care. This study aims to explore older adult care education for (migrant) family care assistants at the European level, highlighting strengths and weaknesses to improve training.

**Methods:**

26 older adult care courses were identified through desk research carried out between December 2023 and March 2024 and analyzed. Information regarding curricula, contents, length, and teaching methods was extracted and compared to identify common and specific characteristics of the training programs.

**Results:**

Findings highlighted an often expensive, unstandardized, and jeopardized offer of older adult care education and the scarcity of training, addressing the emotional, cultural, social, and linguistic needs of MFCAs because learners are not consulted during the course design.

**Discussion:**

Courses should be co-funded by the public administration to ensure access for low-income people, co-designed with the target and embedding host country socio-cultural contents and migration/work legislation. Lessons on dementia, behavioral disorders, cognitive stimulation, and internships at older people’s homes, supervised by the Public Authority, are recommended.

## 1 Introduction

On 1 January 2023, the European population was at 4,488 million people, where 21.3% were aged 65 and over (24% in Italy). This is an increase of 0.2% points compared with the previous year and an increase of 3.0% points compared with 10 years earlier (1). The median age in the EU increased by 2.3 years between 2013 and 2023 (more than 4 years in Italy), reaching 44.5 years in 2023.

With life expectancy, the percentage of older old people with multimorbidity increases, questioning the sustainability of the long-term-care (LTC) systems that are facing an unprecedented shortage of healthcare workers (2, 3).

Due to the insufficient professional care staff and the inability to properly address the increasing demand of home care paired by the willingness of older people for ageing in place (4), many European formal health organizations (hospitals and older adult care facilities, nursing home, etc.) employ caretakers from other countries such as doctors, nurses, and nursing assistants (5). Similarly, in the informal care sector, many families hire foreign care workers for having customized, personalized, and H24 assistance for older people with LTC needs and to address the willingness of older people for aging in place (4). This happens regardless of the welfare and the LTC system (6, 7), but it is common, especially in familialist care regimes, where the family members (mainly women) are expected to play the primary role in ensuring care to dependent (older) relatives in the face of a Government playing a subsidiary role through the provision of monetary transfers (8, 9).

In international literature, foreign care workers with a migration background are called “migrant care workers” (MCWs), identifying both those working in the formal or informal care sectors indiscriminately (37). They are defined as economic migrants, mostly middle-aged women, who move from poorer to richer countries, especially from Central and Eastern Europe to Western Europe, pushed to leave their native countries due to poor wages, poor working conditions, or inability to find a job (10).

In this paper, we focused on foreign care workers hired by private families of older people with LTC needs and called them as “migrant family care assistants” (MFCAs) to distinguish them from the care workers employed in the formal care sector and also to underline the fact that they become part of the household (most are live-in carers), simulating family care ties in the older care recipient’s domestic environment (by taking alive the family obligations care model), to cover the gap in the provision of public, personalized, and continuous home care (11).

In Austria (2017), for example, the overall number of MFCAs was approximately 70,000 (10). In Germany, the number of MCWs ranges between 100,00 and 200,00, mostly from Poland and increasingly from Ukraine (12, 13), where approximately every fifth migrant worker is a household worker.

In Italy, in 2022, lived 621,716 foreign workers (including both cleaners and MFCAs): MFCAs represent 50.2% and come mostly from Georgia, Bulgaria, Ukraine, and Romania (14). These numbers refer to foreign people who have a regular work contract and who can benefit from social, welfare, and healthcare protection measures, even if they are not as advantageous as those available for healthcare workers employed in the formal healthcare. However, it is worth mentioning that, sometimes, MFCAs may not have a regular work contract because the employees do not want to pay the cost of social protection or because the workers already have another and it would be fiscally disadvantageous for them to have two contracts.

As underlined by the European Observatory on Health Systems and Policies (10), the exclusion from legal measures of labor protection and other regulatory laws increases the exposure of MFCAs to the risk of exploitation, which in Italy was worsened by the COVID-19 pandemic, which increased their precarity (15).

Exploitative working conditions and labor rights abuses on MFCAs working in Italy can translate into poor mental health, e.g., a specific depressive disorder among returning MFCAs, known as “Italy syndrome” (16), inequalities in the access to healthcare services (17, 18), and social isolation (19).

Education is widely recognized as a cornerstone for advancing human development, reducing inequalities and empowering individuals, especially among marginalized and migrant populations, thereby serving as a crucial tool to prevent exploitation and promote dignity (20).

Education and training of MFCAs may boost employment, social integration, and improve migrants’ well-being and quality of life (21, 22), especially if implemented by non-profit organizations and promoted by the employment services at the local level (23).

Nevertheless, the educational offer is often jeopardized, non-standardized, and fragmented in many European countries (24). Moreover, the great part of the courses is designed for migrants who want to work in the formal healthcare sector and not in the informal one, and the training does not match several criteria guaranteeing their effectiveness. Moreover, a European common and homogeneous set of appropriateness and effectiveness assessment criteria of the provided education is still missing (25) and should be agreed upon, considering that many immigrants move across the EU countries.

Recent research underlines the need for training specifically designed for migrants, focusing on language, healthcare terminology, and culture of the hosting country (26), and co-designed with the target for combining bottom-up (i.e., the employee’s) and top-down (i.e., the employer’s) vision throughout the decision process in a care setting (25).

Moreover, since migrants represent a vulnerable population group, their social, relational, and emotional needs should be addressed by the courses and not only technical competences to facilitate the learning (23).

In Italy, older courses for (M)FCAs are implemented by private vocational education organizations and by non-governmental organizations that are entitled to provide this kind of education, but are not always certified by the Regional Authority. When the courses are organized by private vocational organizations, their cost is often not affordable by migrants who, very often, face social and economic deprivation.

According to the regional laws, the courses have to last at least 100 h and should often take place during the day: many MFCAs work during the day, and leaving or neglecting care work to attend a training is not an option, as they often have no other resources, such as savings or annuities, for addressing basic needs. In addition, most MFCAs are women, often mothers, without a family network to help them reconcile work, family, and training, so they may be doubly discriminated (10, 21).

Moreover, the lack of a law-based training requirement to be employed as a care worker in private households (unlike other healthcare professionals working in the formal care sector), coupled with the enormous demand for care work, does not make MFCAs motivated to attend courses.

Last but not least, many courses are only in Italian and they do not foresee linguistic or cultural mediation, and this discourages the attendance of many migrants.

Considering the variegated jeopardized, non-standardized, and fragmented offer of care trainings across Europe and the different norms that regulate the education in the informal care sector at the national level, it is important to systematize the knowledge about the characteristics of the courses, e.g., contents, costs, providers, hours of lessons, and style (if online, face-to-face, blended) to boost such an offer and then also promote MFCAs’ human development, quality of life as well as the quality of the care provided to older people. Nevertheless, to our best knowledge, there are no studies focused on this topic. This multiple case study is aimed at covering this gap in knowledge by analyzing 26 older adult care training courses targeted to migrant and no migrant people who are or aspire to be employed as care workers in older people’s households to identify educational curricula, common and prevalent contents, educational styles, and means.

## 2 Materials and methods

The study wants to answer the following research question: Which are the characteristics (contents and modalities) of the selected older adult care training courses for (migrant) care workers in Europe? The study was carried out within the “Aging well in an aging society” project (PNRR PE8 “AGE-IT”), which also aims to develop an e-learning platform for formal and informal caregivers of older people with dementia in Italy. The results of the study analysis will lead to the design of the e-learning platform.

### 2.1 Study design: a multiple qualitative case study

This is a multiple holistic qualitative case study. It was designed as “multiple” (26 cases were analyzed) because of the large variety of training contents and methods. The study was also designed as a holistic case study (27) because many units of meaning were analyzed for every training (e.g., contents, targets, and implementing organizations, as will be deepened in the following paragraphs).

Finally, the qualitative approach was chosen to understand the complexity of the topic and speculate its impact on learners and care recipients (28, 29).

This study adopted a multiple-case qualitative design, as outlined by Yin (27), to explore and compare older adult care training for migrants and non-MFCAs across Europe. A total of 26 training initiatives were treated. Each case was independently analyzed based on a shared template (Table 1), focusing on key dimensions such as curriculum content, delivery mode, cost, target group, and certification.

**Table 1:** Template for the analysis of the older adult care training for (migrant) family care assistants.

Units of analysis	Item description	Contents
Abstract	This item is aimed at briefly describing the initiative by summarizing implementing organization; country; objectives; contents; language.	Descriptive text
Target and participants’ admission criteria	This item is aimed at identifying the main target group and the criteria for the admission of the participants	Descriptive text
Implementing/delivering organisation	This item is aimed at reporting the name of the organization implementing the training	Name of the organization that funded/designed/implemented the training
Public funding	This item is aimed at highlighting the public nature of the organization implementing the training	YES/NO
Country	This item identifies the country where the course is implemented	NAME
Training timeframe	This item is aimed at reporting the length of the training	Descriptive text
Main objective	This item is aimed at reporting the primary and the secondary (when present) objectives of the training	Descriptive text
Contents of the training: modules and lessons	This item synthetizes the contents of the lessons	Descriptive text
Way of delivery	This item highlights the style of training delivering	In group/ Individually/ Mixed/ Tutoring foreseen
Medium	This item wants to indicate the medium used for the training	Online/In person/Blended
Co-designed and details if available	This item is aimed at highlighting if the co-design approach is common in trainings for informal caregivers	Yes/No If yes, descriptive text where information available
Analysis of learning needs	This item is aimed at verifying if the analysis of learning needs has been done	Yes/No
Learning outcomes assessment	This item is aimed at verifying if the learning outcomes are assessed	Yes/No
Certification provided	This item is aimed at explaining the certification process	By whom/where is it valid (regional, national, EU level)
Re-funding of participants	This item wants to understand if the participants receive any reimbursement for attending the training	Yes/No If yes, how much
Web link	This item reports the web link of the initiative for deepening	URL
Scientific publication(s)	This item reports any scientific publication, green, white paper on the experience of the training	References

According to Yin’s methodology, a within-case analysis was conducted for each training program, followed by a cross-case synthesis to identify patterns, similarities, and differences. The use of a replicable analytic framework ensured consistency across cases, and the holistic approach allowed for the exploration of multiple dimensions within each training (e.g., contents, targets, implementing organizations), as will be deepened in the following paragraphs.

The researchers followed five steps: (a) the definition of the research design, i.e., the setting of research question and of the research keywords; (b) the preparation of the template for the data collection; (c) the data collection, i.e., the review of the trainings available online; (d) data analysis, i.e., the detailed description of every training and the analysis of the contents and methods; (e) the interpretation, i.e., the reporting of the meaning of the cases. The trainings were identified through desk research that was held between December 2023 and March 2024.

The Authors followed the Standards for Reporting Qualitative Research (SRQR) guidelines to ensure transparency and reproducibility in their qualitative research.^1^

It is worth mentioning that this study did not want to map the existing training on older adult care in Europe, but it was aimed just to analyze a selection of them to give an overview of the characteristics of the available offer of education in the field.

### 2.2 Inclusion criteria

Due to the scarcity of older adult care courses specific to migrants employed in the informal care sector, in a first moment, the research was focused on older adult care courses offered to adults willing to work as family care assistants in Italy, regardless of their migration background. Nevertheless, special attention was paid to the courses specifically targeted to migrants and refugees or that included some lessons. In a second moment, the authors extended the research to other European countries characterized by long-standing older adult care emigration (e.g., Romania and Ukraine) and immigration flows (e.g., Italy and Germany)—the so-named care drain—to compare the Italian offer to that from abroad.

The following keywords were chosen to surf the Google search engine: “migrants”; “refugees”; “MCWs” (because this definition incudes care workers both in the formal and informal care setting); “family care assistants”; “education”; “training”; “courses”; “elder(ly) care”; “long-term care.”

The trainings that were included are as follows: aimed at people who wanted to work in the informal care sector; focused on older adult care; still ongoing, and implemented in the main European “care drain” countries. The trainings that were excluded are as follows: were finished; were aimed at training people on working in the formal health care sector, e.g., courses for nurse assistants, because our research was focused on the education of people working for older people with LTC needs living at home.

The search was implemented in German, Italian, English, Czech, Polish, and Romanian languages. Czech, Polish, and Romanian languages were chosen because most home care workers in Italy come from Eastern Europe (6, 7), and German was chosen because German and Austrian families are used to employing migrant care assistants. The search for keywords in Czech and Romanian was carried out with the support of the Google automatic website translator.

Initiatives were included if they were carried out since January 2023 and were still ongoing in March 2024 or planned for a few more months. The information was updated till December 2024. Thus, it is possible that at the moment of publication of this paper, some trainings may be replaced or updated by the providing organizations.

### 2.3 Analysis

A template for the description of the initiatives was designed (Table 1), whose analysis units were chosen based on the literature on the topic, mainly reported in the Introduction.

The information available on the websites of the implementing organizations was carefully read by two researchers to extrapolate the information useful to fill in the template for the analysis. Every case was described in detail based on the collected data, and the data matching the selected topics (Table 1) were analyzed. Both intra- and intercase analyses were performed.

To ensure the study quality, a prolonged and persistent reading of the training between December 2023 and November 2024, a constant debriefing and checking within the research team, the audit trail, and a thick description of every training enclosed in the analysis were carried out (30).

## 3 Results

The desk research identified 26 older adult care training initiatives in total: 1 from the Czech Republic (CR), 4 cross-national (CRS), 2 from Germany (DE), 1 from Ireland (IR), 12 from Italy (IT), 2 from Poland (PL), 2 from Romania (RM), and 2 from the United Kingdom.

Table 2 reports, in an aggregated way, the characteristics of the training (i.e., the unit analysis) by country.

**Table 2:** Characteristics of trainings by country.

Country (N of trainings)	CR (1)	CRS (4)	DE (2)	IR (1)	IT (12)	PL (2)	RM (2)	UK (2)	Total (26)
Provider									
Public vocational/educational organizations/NGOs	0	2	2	0	2	0	0	1	7
Private vocational/educational organizations	1	2	0	1	10	2	2	1	19
Cost									
Free	0	2	2	0	2	0	0	1	7
Co-covered by the public	0	0	0	0	1	0	0	0	1
<500€	1	1	0	1	0	2	0	0	5
501–999€	0	0	0	0	3	0	0	0	3
≥1.000€	0	0	0	0	3	0	0	0	3
Not available	0	1	0	0	3	0	2	1	7
Co-designed	0	2	0	0	2	0	0	0	4
Multilanguage course	0	3	0	0	0	0	0	0	3
Designed specifically for migrants	0	1	1	0	2	0	0	0	4
Way of delivery									
Online	0	2	0	1	1	1	0	2	7
In person	1	1	0	0	8	0	2	0	12
Blended	0	1	0	0	3	0	0	0	4
Not specified	0	0	2	0	0	1	0	0	3
Lessons setting									
Individually	0	2	0	1	0	1	0	0	4
In group	1	2	0	0	12	0	2	2	19
Not specified	0	0	2	0	0	1	0	0	3
Training curriculum									
Generic	0	1	1	1	5	0	0	0	8
Detailed	1	3	0	0	5	1	2	1	13
Not specified	0	0	1	0	2	1	0	1	5
Contents									
Dementia	1	0	0	0	1	0	0	1	3
Host country language	0	1	1	0	0	0	0	0	2
Hygiene and personal care	1	4	2	1	12	2	2	2	26
Health and safety of the cared for person	1	4	2	1	12	2	2	2	26
Emergency management	0	0	0	0	5	1	2	0	8
Communication	0	1	0	0	4	0	2	0	7
Legal aspect of domestic work	0	0	0	0	3	0	1	0	4
Nutrition	0	2	0	0	4	1	0	0	7
Self-awareness and health of the caregiver	0	0	0	0	0	0	0	1	1
Internship (Yes)	0	1	0	0	1	0	0	0	2
Length of training (expressed in hours)									
<50	1	0	0	0	0	0	0	0	1
51–99	0	0	0	0	1	2	0	0	3
100–199	0	3	0	0	3	0	0	0	6
200–399	0	0	0	0	5	0	2	0	7
400–600	0	0	1	0	2	0	0	0	3
Length of training (expressed in years/months/weeks)	0	0	1	1	1	0	0	2	5
Length not specified	0	1	0	0	0	0	0	0	1
Certification (Yes)	1	4	2	1	12	2	2	2	26

Values in bold indicate the key analytical dimensions.

A total of 19 courses out of 26 were provided by private vocational education organizations, and only seven are free (two cross-national, two in Germany, two in Italy, and one in the UK). The most expensive courses are in Italy, where three of them are between 500 and 999€, and the other three are over 1,000€.

Only four courses are co-designed (two in Italy and two cross-national), three are multi-language, three cross-national, and four are specifically designed for migrant learners.

The most common way of lesson delivery is in person (12 cases), and the preferred setting is in a group (19 cases).

Half of the courses analyzed provided a detailed training curriculum by specifying lesson by lesson, while the remaining ones limited to give information on large thematic areas or did not specify contents at all. According to the available data, cross-nationally, the most covered contents are “Hygiene and personal care,” “Health and safety of the cared for person,” “Emergency management,” and “Nutrition.” The most uncovered are “Caregiver’s self-care,” “Host country language,” and “Dementia.” The length of the training is expressed in different ways, i.e., in hours, months, weeks, and years, so it is difficult to identify a trend cross-nationally or calculate the minimum number of hours of education, either in the countries or cross-nationally.

The Supplementary Table 2.1: “Detailed trainings codes by analysis unit” reports which courses have the specific characteristics described in the analysis units, and Supplementary Table 3 “older adult care trainings description” provide a deeper case-by-case analysis. While Supplementary Table 2.1 is easy to read and needs no further explanation, the length of Supplementary Table 3 prompts us to put it in the supplementary materials, but its complexity required that its contents be summarized below to better understand the results of the analysis carried out. Thus, looking at this table, 10 out of 12 trainings delivered in Italy were provided by private vocational education companies, and they are out-of-pocket (IT1, IT2, IT3, IT4, IT5, IT6, IT7, IT8, IT9, and IT10) (Supplementary Table 3). Their cost ranges between 700€ and 1.500€, and the hours of training are between 100 and 900. Two trainings are provided by non-governmental organizations (NGOs) (IT11, IT12), of which only one training is out-of-pocket (it costs 700€) (IT1). All the Italian courses last between 64 to 250 h. Regardless of the length, all trainings foresee a certification delivered by the competent authority (e.g., the Regional Council), and the common contents are: hygiene, first aid/management of the emergency, movement of the disabled person, nutrition, and communication. The lectures are delivered in Italian, and just two trainings (IT11 and IT12) are targeted specifically at migrants and are totally free. One is targeted to foreigners and, in particular, Ukrainian refugees, and is aimed at increasing older adult care knowledge and competences (IT11). The other one is aimed not only at education but also at reception, social inclusion, and employment of Ukrainian citizens with refugee status (IT12).

Concerning the 10 trainings held in Europe, seven are promoted by private vocational education organizations (CR1, PL1, PL2, RM1, RM2, IR1, and UK2) and three by public vocational education organizations (DE1, DE2, and UK1). In Germany, the two analyzed initiatives (DE1, DE2) are designed for MFCAs, are totally free, and last about 2 years. DE1 focuses on improving migrants’ German language, and DE2 addresses migrants with a German language level A2. Both courses are held in public schools and state-approved or state-recognized vocational schools for older adult care assistance with the aim of providing professional knowledge, skills, and abilities for the assistance of sick older people.

In contrast to German courses, the trainings implemented in Eastern Europe are mainly carried out privately, their cost ranges between 70,00€ and 120,00€, and their length is between 24 and 360 h in total, often a mix of theoretical and practical learning. The training held in the Czech Republic (CR1) is paid out-of-pocket, provided by the private Military University of Prague, and accredited by the Ministry of Education and Culture. It is about assistance focused on the needs of older people, acquiring theoretical and practical knowledge focused on the concept of LTC, without attention to migrants and refugees.

In Poland, the two reported older adult care courses are provided by private vocational education organizations (PL1, PL2). The lessons are carried out in groups and in person with a final recognized certification.

The two courses from Romania (RM1, RM2) are provided by private vocational education organizations. They are aimed at acquiring the knowledge and skills necessary to successfully practice the activity of caring at home. They are paid by the trainees, but the cost to participate is not specified. Both consisted of 360 h and are not specific to migrants or refugees.

Noteworthy, courses organized in Eastern European countries are designed for citizens (most women) who want to emigrate to Central and Southern European countries to work as MFCAs, strengthening the “care drain” trend.

Two out of three older adult care trainings identified in Ireland and the UK are provided by private vocational adult education organizations without any attention to migrants or refugees. The cost is not reported on the website as it is made available upon request. The length is reported in weeks, months, and/or hours, and it is not possible to compare the different offers and understand an average number of training hours. The Irish training (IR1) is aimed at developing skills, knowledge, and understanding of the role of a care assistant, and promoting good practice with an online presence of 16 weeks.

The training initiatives from the UK aim to provide essential principles of older adult care and train students in supporting vulnerable people, exploring difficulties and challenges while caring for older adults. The training UK2 includes a lesson on the caregiver’s self-care.

At the cross-national level, two courses are managed by private vocational education providers (CRS1 and CRS3). The length ranges between 75 and 100 h of lessons. Three out of four (except for CRS3) are free for the trainees since the curriculum design and the course implementation are funded by the Erasmus+ Programme. Except for CRS3, whose lessons are provided only in English, the trainings of this subgroup are available at least in four languages. The length varies from course to course, and in three cases, it is not declared on the website. All these courses are targeted at migrants.

CRS3 is focused on migrants and refugees from the Middle Eastern and African countries in Europe with the objective of training them on older adult care to boost social inclusion, quality of life, and employability. A training unit on information and communication technology is what mainly distinguishes CRS4 from other training, both cross-nationally and at the European level.

Concerning the learning style and media, only seven of the reported trainings are implemented exclusively online (IT4, PL2, IR1, UK1, UK2, CRS1, and CRS4) and, noteworthy, since e-learning platform are never mentioned, we assume that the distance learning only consists on lessons provided through video-conference that allow synchronous lessons, but that cannot work as educational materials repository, asynchronous lessons and private forum for learners and teachers.

Table 2 shows the topics most covered by the lessons, but in Supplementary Table 3, we can identify some country peculiarities. In the Italian courses, for example, attention is paid to communication and inter-personal relationships between the caregiver and the caretaker (IT1, IT2, IT5, IT8, IT9, IT10) and to the legal aspects of the care work (IT1, IT8, IT9), more than in courses held in other countries. Conversely, the Polish courses seem to be more oriented to the nursing activities, e.g., measurement and analysis of life parameters and protection against infection, while Romanian trainings try to integrate humanistic and nursing topics.

## 4 Discussion

This study was aimed at analyzing the contents and methods of older adult care training programs for migrants working or aspiring to work as family care assistants of older people with LTC needs.

### 4.1 Strengths and weaknesses of the current offer of older adult care education

The study confirms a jeopardized, not standardized, unclear offer of older adult care education in the informal care sector (24) and the limited availability of older adult care training programs in Europe specifically targeted to people with a migration background and including lessons on the hosting country language (19).

As we could expect, the trainings implemented in Eastern European Countries are not targeted at migrants and refugees but to citizens who plan to emigrate to Western, Central, and Southern European countries to work as MFCAs. In light of this, such courses should focus more on self-care and resilience, so that people who leave their native country to become MFCAs can better cope with the separation from their loved ones and have the tools to prevent and cope with mental health issues, e.g., the so-called “Italy syndrome” (16).

Across Europe, the courses are mostly paid, and this is excluded from the attendance of most migrants, who often experience economic difficulties due to the unfair working conditions and low salaries (9).

Mirroring the literature (25), few trainings are co-designed and are based on the identification of the target’s needs. Noteworthy, those initiatives exploring learners’ needs focused only on the learning ones, neglecting social and emotional needs.

As most trainings are not targeted to people with a migration background, they do not embed contents on the host country culture, social representations of ageing, disability, care, illness and sickness nor the social values that can shape daily care activities (23) and migration legislation as instead recommended by the literature (25). Another undercover topic is dementia: only three courses mention Alzheimer’s Disease or dementia within the topics of the lessons (IT1, CR1, and UK2). Educating MFCAs on the neuropsychiatric symptoms of Alzheimer’s disease and dementia, e.g., behavioral symptoms, and on how to manage them it is of crucial importance in light of the fact that dementia represents one of the main reasons for LTC demand globally (31). Moreover, when Alzheimer’s disease and dementia are included in the lessons, the topic of cognitive stimulation (e.g., simple and enjoyable activities aimed to stimulate thinking and memory, word games, puzzles, music, and creative practical activities) is completely under-represented. This may depend on the wrong belief that an (M)FCA has to deal mainly, if not exclusively, with the practical assistance, e.g., cleaning, feeding, and bedsore prevention. Conversely, the (M)FCA should be meant as a professional who embodies nursing, social, and psychological competences because “assisting is not enough,” paraphrasing Pasquinelli and Ruffini’s essay (32).

The topic of communication with the older person is largely covered, but the communication of the MFCAs to the healthcare professionals and to the family carers is rather limited.

Noteworthy, lessons on self-care and resilience are not common among the analyzed trainings, despite being well known how serious the consequences of care burden can be: intense emotional distress, fatigue, sleep disorders, and difficulty maintaining the energy needed to provide care (33).

Only the case from the Czech Republic (CR1) embeds a lesson on how to take care of the spiritual well-being of the care recipient, and only one of the two cases from Poland (PL1) trains the family care assistants on how to accompany the assisted person to death. None of the reported cases includes education or information on home palliative care.

Based on the information available online, only two trainings include an internship (IT8 and CROSS2). When the internship is mentioned, it is not described in detail. It is not specified where it is held, whether in hospital wards, in care or elder facilities, or if it puts the trainees in contact with older patients, and which activities the trainees can practice.

In light of these results, we therefore speculate that the courses analyzed do not fully address the educational and socio-emotional needs of MFCAs.

As for the learning media, distance-learning courses may be convenient because they are accessible from anywhere and at any time. This is important for migrant trainees because they often only use public transport and work several jobs in different parts of the city, and so they may face difficulties in reaching the training place. At the same time, however, access to both a computer and a stable internet connection, language barriers, and the need for socialization and peer comparison of migrants can represent obstacles for their engagement in the educational process, and then make distance learning less effective.

In a few cases, the initial learning objectives and the chances of employment after the attendance of the course were clearly reported. Moreover, the criteria for the evaluation of the learning outcomes and the delivery of the certifications are not clear in most training descriptions.

Finally, looking at the courses delivered in Italy, we identified a lack of clarity that starts right from the naming of the target: some courses are for “family assistants,” others for “basic care workers,” and others for “family care assistants.” Unfortunately, we are not able to grasp the same nuances in other languages as the research was not carried out by Czech, Romanian, Polish, etc., native speaker researchers, as reported below.

### 4.2 Lesson learned: practical, policy, and research suggestions

Despite the described limitations, the study collected several information that can shape the design and implementation of future trainings in older adult care. A training for MFCAs should be designed with the target of education through consultation sessions planned for identifying not only educational but also (if not mainly) emotional, economic, and relational needs of the trainees. The co-design should also concern times and style of learning for agreeing weekly day and time and the percentage of face-to-face lessons that migrants can realistically attend. The consultation of the trainees can be made through in-depth interviews to know and understand learners’ past life experiences, future plans, and expectations that the training may boost and support. The course should be co-funded by the public administration and co-paid by migrants (i.e., not totally free) to be fully accessible also by people experiencing economic restrictions and, at the same time, to elicit their sense of duty and responsibility. Moreover, it is recommended that future trainings in older adult care foresee a practical experience. Since they are targeted to (M)FCAs who will work at home, it would be better if the internship were at the home of older people. The internship should be supervised by the public authority (e.g., the Municipality or the Region) that may guarantee that the principle of equity is respected and organized in full cooperation with training providers, voluntary organizations, law enforcement, and insurance companies to ensure safety and privacy of both trainees and older people.

The analysis of the cases calls for future trainings focusing more on cultural and social representations of care and ageing, migration legislation, techniques for managing behavioral disorders and stimulating people with dementia, self-care and resilience, communication with family carers, home palliative care, and till the end of life.

At the policy level, regional and municipal administration should attract funds for implementing the courses and make the attendance of an older adult care training mandatory, and help the relatives of older people in need of care find substitutes for the main MFCA attending the course. To ensure full accessibility to education, policy measures are needed that can provide migrants with devices and internet connections for attending distance-learning courses.

Furthermore, it is recommended that the educational offer for migrants in older adult care is regulated by local/regional authorities according to clear guidelines at the European level. The latter would guarantee the harmonization of educational contents and criteria of evaluation across Europe to boost the recognition of the acquired competences from one country to another in case the migrants move across Europe.

Despite the large employment of MFCAs and the pivotal role they play in the provision of home care across Europe, education and training are still an under-covered topic in the international scientific literature. Most studies indeed underline the impact of caregiving on MCWs’ mental and physical health (16, 34), others underline the unfair working and social condition they experience (35, 36), but few studies focused on the quantity and quality of the educational offer for migrant people who are working or want to work as carers of older adults with LTC needs. It is therefore recommended that future pre-post and longitudinal studies shed light on the characteristics of the training as well as on the impact they have on the employability, quality of life, social inclusion of MFCAs, and on the quality of the care received by older people.

## 5 Strengths and limitations of the study

In this perspective, this multiple case study contributes to an increase in the knowledge about the main characteristics of the available older adult care education for migrants and no MFCAs for improving future trainings contents, style, and means.

The main strength of this research is its rapidity, and the second lies in the fact that the analysis criteria were chosen based on the literature on the topic, and then allowed to shed light on the aspects to improve further educational programs for migrants and refugees.

The study also presents some limitations. The first is the incomplete provision of information due to the lack of transparency of several private trainings concerning costs and curricula that were accessible just after logging into the platform, giving personal details, and applying for admission. Moreover, the use of only some languages and the use of online translators for identifying the trainings offered in non-English-speaking countries has limited the findings.

## 6 Conclusion

European LTC systems need the work of migrant workers, especially in the informal care setting, to provide personalized home care for the growing number of dependent older people. Care workers with a migration background need to be trained to be able to provide quality care and improve their socio-economic status and human development. The available trainings are often very expensive and do not consider language difficulties, cultural characteristics, and socio-emotional needs of migrants. Future older adult care trainings should be co-designed on the mentioned needs; include guidelines on dementia care and home palliative care; consider both native and hosting country cultural issues; and be linguistically, economically, and practically accessible.

## Data Availability

The original contributions presented in the study are included in the article/Supplementary material, further inquiries can be directed to the corresponding author.

## Footnotes

^1^ https://www.equator-network.org/reporting-guidelines/srqr/
