# Supplementary material for: Educational offer in older adult home care for migrant family care assistants: results from a multiple qualitative case study
Source: Front Public Health. 2025 Sep 12;13:1621071. doi: 10.3389/fpubh.2025.1621071 (PMC12463628; doi:10.3389/fpubh.2025.1621071)
Supplement: Supplementary file 1 [file Table_1.DOCX]

Supplementary Material

## Table 2.1 Detailed trainings codes by analysis unit

| Characteristics | Training codes |
| --- | --- |
| Provider |  |
| Public vocational/educational organizations/NGOs | IT11, IT12, DE1, DE2, UK1, CRS2, CRS4 |
| Private vocational/educational organizations | IT1, IT2, IT3, IT4, IT5, IT6, IT7, IT8, IT9, IT10, ,, CR1, PL1, PL2, RM1, RM2, IR1, UK2, CRS1, CRS3 |
| Cost |  |
| Free | IT11, IT12, DE1, DE2, UK1, CRS2, CRS4 |
| Co-covered by the public | IT3 |
| <500€ | CR1, PL1, PL2, IR1, CRS3 |
| 501-999€ | IT1, IT2, IT10 |
| ≥1.000€ | IT8, IT8, IT9 |
| Not available | IT4, IT5, IT6, RM1, RM2, UK2, CRS1 |
| Co-designed: consultation with training beneficiaries and stakeholder | IT8, IT12, CRS2, CRS4 |
| Multilanguage course | CROSS1, CROSS2, CROSS4 |
| Designed for migrants | IT11, IT12, DE1, CROSS 2 |
| Way of delivery |  |
| Online | CRS1, CRS4, IR1, PL2, UK1, UK2 |
| In person | CR1, CRS3, IT1, IT3, IT6, IT7, IT9, IT10, IT11, IT12, RM1, RM2 |
| Blended | CRS2, IT2, IT5, IT8 |
| Not specified | DE1, DE2, PL1 |
| Lessons setting |  |
| Individually | CRS1, CRS4, IR1, PL2 |
| In group | CR1, CRS2, CRS3, IT1-IT12, RM1, RM2, UK1, UK2 |
| Not specified | DE1, DE2, PL1 |
| Training curriculum |  |
| Generic | CRS4, DE2, IR1, IT2, IT4, IT5, IT6, IT7 |
| Detailed | CR1, CRS1, CRS2, CRS3, IT1, IT8, IT9, IT10, IT11, PL1, RM1, RM2, UK1 |
| Not specified | DE1, IT3, IT12, PL2, UK2 |
| Contents |  |
| Dementia | IT1, CR1, UK2 |
| Host country language | DE1, CRS2 |
| Hygiene and personal care | All |
| Health and safety of the cared for person | All |
| Emergency management | IT1, IT4, IT6, IT9, IT10, PL1, RM1, RM2 |
| Communication | IT1,IT5, IT8, IT9, RM1, RM2, CRS2 |
| Legal aspect of domestic work | IT,1, IT8, IT9, RM1 |
| Nutrition | IT4,IT6,IT9, IT10, PL1, CRS1, CRS3 |
| Self-awareness and health of the caregiver | UK2 |
| Internship (Yes) | IT8, CRS2 |
| Length of training (expressed inhours) |  |
| <50 | CR1 |
| 51-99 | IT11, PL1, PL2 |
| 100-199 | IT3, IT5, IT6, CRS1, CRS2, CRS4 |
| 200-399 | IT1, IT7, IT8, IT10, IT12, RM1, RM2 |
| 400-600 | IT2, IT9, DE1 |
| Length of trainings (expressed in years/months/weeks) | DE2, IR1, IT4, UK1, UK2 |
| Not specified | CRS3 |
| Certification (Yes) | All |

## Table 3 Elder care trainings description

| **Training code** | **Provider (Private/Public) and cost** | | | | **Contents** | **Cost** | **Specific for migrants**  **(Yes/No)** | | **Way of delivery** | **Hours of training** | | **Certification (if yes by whom)** |
| --- | --- | --- | --- | --- | --- | --- | --- | --- | --- | --- | --- | --- |
| IT1 | Private  Social cooperative | | | | -Hygiene and personal care  -Handling and lifting techniques  -Community services  -Management of emergencies  -Relational psychology  -Communication techniques with Alzheimer's patients  -Legal aspects of domestic work  -Health and safety of the cared for person | Out-of-pocket  700,00€ | No | | In group  In person | 220 hours | | Yes, by the Tuscany Region |
| IT2 | Private VET organization | | | | -Pedagogy  -Relational Psychology  -Social Integration  -Hygiene and prophylaxis  -Health needs and problems  -Personal care  -Personal and environmental hygiene  -Comfort and safety | Out-of-pocket  700,00€ | No | | In group  Blended | 600 hours | | Yes, by the Tuscany Region |
| IT3 | | Private  Social cooperative | | | Not specified | Co-covered by public funding | No | | In group  In person | 160 hours | | Yes |
| IT4 | | Private VET organization | | | -Elements of anatomy and physiology  -First aid and hygiene  -Nutrition  -Home economics  -The elderly and disabled  training | Out-of -pocket  Price details under request | No | | In group  Online | 6 months 100 hours of stage | | Yes |
| IT5 | | Private VET organization | | | -Social services and assistance  -Communication and relations  -Care and accompaniment  -Environmental management and security | Out-of -pocket  Not specified | No | | In group  Blended  (34 h online and 34 h in-person) | 100  hours: -68 hours of theoretical lessons  -10 hours of practice -22 hours of stage | | Yes |
| IT6 | | Private VET organization | | | -Psychology  -Hygiene  -Nutrition  -Movement and motor management  -Dressing  -First aid  -Legislation and professionalism | Out-of -pocket  Not specified | No | | In group  In person | 160 hours | | Yes |
| IT7 | | Private VET organization | | | -Hygiene  -Care  -Information and communication technologies | Out-of -pocket  1000,00€ | No | | In group  In person | 200 hours (130 theory and exercise + 64 hours of stage + 6 hours for exam) | | Yes |
| IT8 | | Private VET organization | | | - Elements of communication and interpersonal relationships in care work  - Legal elements and territorial network services  safety and health promotion  - Personal assistance and care  - Tools for care of environments, organization and monitoring of your work  - Key and transversal skills  - Individual and group accompaniment/orientation activities  - Collective orientation  - Individual orientation | - Out of pocket: 1520€;  - Public funding for unemployed people | No | | In group  Blended | 600 hours (370 hours of theoretical-practical activities, of which 70 hours of FAD and 230 hours of cognitive and applicative internship) | | Yes |
| IT9 | | Private VET organization | | | - Elements of relational psychology and interpersonal communication techniques  -Orientation to the territory and use of services  -Social Support and promotion  -Monitoring and evaluation of own work  -Hygiene and personal care  -Elements of nursing and handling techniques  -Elements of first aid  -Needs analysis  -Safety in the workplace  -Elements of professional  -Ethics, legislation, labor law and contracts  -Elements of dietetics and nutrition  -Food preparation and serving  elements of home economics | Out-of-pocket 1.500,00€ | No | | In group  In person | 600 hours | | Yes, by the Tuscany Region |
| IT10 | | Private VET organization | | | -Ethics and deontology  -Direct assistance in environmental hygiene and domestic help  -Health legislation  -Elements of hygiene  -Mobility assistance techniques  -Elements of nutrition  -Education  -Helping relationships  -Psycho-relational factors  -First aid officers  -Fire-fighters low risk | Out-of-pocket  900,00€ | No | | In group  In person | 200 hours | | Yes, by SardiniaRegion |
| IT11 | | NGO | | | -Care and cleaning of the home-cooking  -Meal preparation  -Meal delivery-help relationship with the elderly person-care  -Hygiene  -Support in walking management of emergencies  -Professional ethics | Free | Yes | | In group  In person | 64 hours | | Yes |
| IT12 | | NGO | | | Not specified | Free^^[[1]](#footnote-1)^^ | Yes | | In group  In person | 250 hours | | Yes, by Campania Region |
| DE1 | | | Public VET organization | Not specified | | Free | Yes | Not specified | | | Two years:  -1440 hours theoretical and practical lessons  -1600 hours practical training | Yes |
| DE2 | | | Public VET organization | -German language  -Care and nursing of older people and people in need of care | | Free | No | Not specified | | | One year  -720 hours of theoretical and practical instruction  -850 hours of practical training | Yes |
| CR1 | | | Private  Military University of Prague (private university) | -Long-term care for older people  -Methods of working with seniors aimed at supporting contact, activity, participation and dignity  -Comprehensive social care for a high-risk client  -A comprehensive activation program aimed at older people  -Caring for the client’s spiritual needs  -Training of basic techniques in manipulation and activation with older people | | Out-of-pocket  3.000,00 CZK (about 120€) | No | In group  In person | | | Three days:  -16 hours theoretical part  -8 hours practical part | Yes, by Ministry of Education |
| PL1 | | | Private VET organization | -The role and tasks of the care assistant  -Elderly diseases, patient observation, measurement and analysis of parameters  -Protection against infection, disinfection, sterilization  -Principles of proper nutrition for sick, elderly and disabled people  -Physiological and physiological aspect of ageing  -Attitude towards the feelings and behavior of the person being cared for  -Prevention of pressure ulcers and the effects of long-term immobilization  -Problems of care for disabled patients  -Care of older patients  -Rehabilitation process  -First aid in geriatrics  -Caring for the dying and accompanying death  -Selected issues of pharmacotherapy | | Out-of-pocket  999 PLN (235,55€) | No | Not specified | | | 60 hours | Yes |
| PL2 | | | Private VET organization | Not specified.  The training contents are available only after the payment of the course fee | | Out-of-pocket  300,00 PLN ( about 70,00€) | No | Individually  Online | | | 80 hours | Yes |
| RM1 | | | Private VET organization | -Supervision and care of older people at home  -Communication and interpersonal relations  -First aid knowledge  -Services adjacent to home care  -Labor law knowledge  -Practice module | | Out-of-pocket  Not specified | No | In group  In person | | | 360 hours:  -120 hours of theory  -240 hours of practice | Yes |
| RM2 | | | Private VET organization | -Communication at work  -Application of NPM and PSI  -Ensuring hygienic and sanitary conditions  -Ensuring professional development  -Completing the care record of the person assisted  -Managing allocated resources  -Planning daily activities  -Providing hygienic care for the assisted person  -Giving first aid to the person assisted  -Ensuring the comfort of the older assisted person  -Assisting with feeding and food management  -Training/ developing skills in the assisted older person  -Mobilizing and transporting the older assisted person  -Mobilizing and transporting the assisted immobile person  -Observing and applying medical prescriptions  -Monitoring the health of the assisted person | | Out-of-pocket  Not specified | No | In group  In person | | | 360 hours | Yes, by the Ministry of Labor and the Ministry of education |
| IR1 | | | Private VET organization | -Patient understanding  -Assistance skills  -Ageing process  -Working with older people  -Care of older people with special needs  -Treatment metodologies | | Out-of-pocket  259,00€ | No | Individually  Online | | | 16 week:  8 weeks per module | Yes |
| UK1 | | | Public VET organization | Not specified. | | Free | No | In group  Online | | | 6 weeks | Yes |
| UK2 | | | Private VET organization | -Dealing with and caring older people  -Risk Factors  -Safeguarding the health of the older person  -Caring for older person diagnosed of dementia  -Top outdoor elderly activities  -The basics of caring for the bedsores  -Mental health basics  -Effective caring for your elderly  -A stressful job  -Healthy relationships  -Listening to your parents even now  -The caregiver enemy no 1  -Taking care of yourself as well as the older vacation and respite | | Out-of-pocket  Not specified | No | In group  Online | | | 18 hours | Yes |
| CRS1 | | | Private VET organization | -Introduction to home care  -Working with older people  -Working with children  -Working with people with development disorders  -Working with people with physical disability  -Working with mentally ill elderly people  -Nutrition and meal preparation  -Personal care  -Family financial administration  -Safety and injuries prevention  -Personal care and hygiene | | Out-of-pocket  Not specified | No | Individually  Online | | | 75 hours | Yes |
| CRS2 | | | Public and private VET organisation and research institute/universities in different EU Countries (Cyprus, Greece, Italy, Portugal) | Language of the host countries (Italian, Greek and Portuguese)  Training on elder care:  -Ageing and neurocognitive disorders  -Communication skills and competences  -Person-centred care and strategies  -Nursing care and practices | | For free  (Erasmus+ funding) | Yes | In group  Blended: in person and online  (through the e-learning platform) | | | 100 h:  78 face to face lessons + 22 hours of internship | Yes  (in Italy by Marche Region) |
| CRS3 | | | Private VET organization | -Understanding ageing  -Communicating with senior  -Infection control  -Understanding and monitoring vital signs  -Nutrition and hydration  -Simple activities and exercises for seniors  -Assisting to serve oral medication to seniors  -Managing emergencies  -Day-to-day care  -Toileting  -Basic skin care  -Feeding  -Personal hygiene, bathing, grooming and dressing  -Transferring, positioning and mobility  -Fall prevention | | Out-of-pocket  $400-$500 | No | In Group  In person | | | Not specified | Yes |
| CRS4 | | | Public consortium.  Public and private VET organisations and research institutes/universities in different EU Countries (Cyprus, Greece, Italy, Portugal) | Two curricula available  Curriculum n.1  a) A-typical Elderly Caregivers  b) Elderly Care Sectoral Providers  Curriculum n. 2: Elderly Care Sectoral Providers | | For free  (Erasmus+ funding) | No | Individually  Online | | | 46 h | Yes |

**
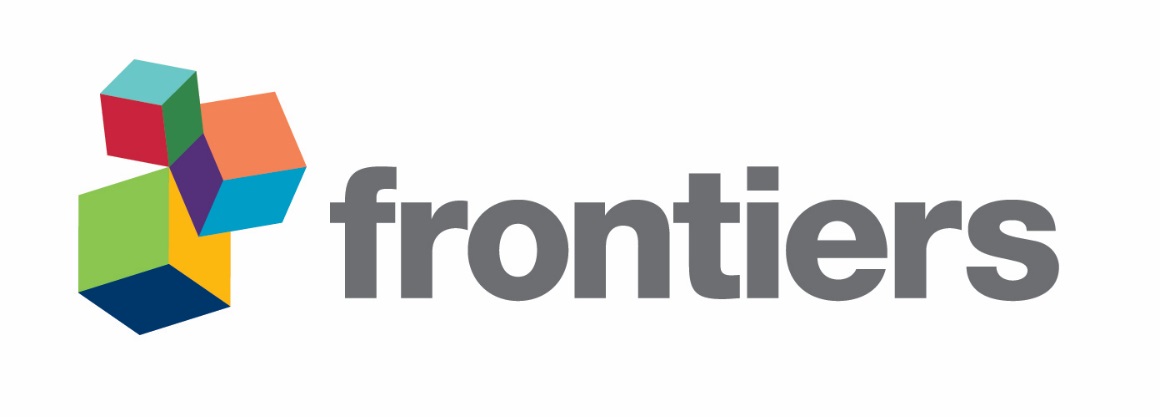
**

1. Those who adhere to the Measure will receive an attendance allowance of €. 3,50 for each hour of lesson followed (financed by Forma.Temp) and, upon achievement of the Certificate, assigned by Generazione Vincente Academy, the reimbursement of any expenses incurred for board, lodging and transport in compliance with the maximum amount provided. An additional allowance of €. 1.000,00 "UNA TANTUM" (financed by Ebitemp) is possible, upon completion of the first training cycle, at the participant's request. [↑](#footnote-ref-1)
